# Atomic reconstruction induced by uniaxial stress in MnP

**DOI:** 10.1038/s41598-023-40806-1

**Published:** 2023-08-23

**Authors:** Tatsuya Kozawa, Masayoshi Fujihala, Takeru Uchihara, Setsuo Mitsuda, Shin-ichiro Yano, Hiromu Tamatsukuri, Koji Munakata, Akiko Nakao

**Affiliations:** 1 Department of Physics, Faculty of Science, Tokyo University of Science, Shinjuku, Tokyo, 162-8601 Japan; 2 Advanced Science Research Center, Japan Atomic Energy Agency, Tokai-mura, Ibaraki, 319-1195 Japan; 3 National Synchrotron Radiation Research Center, Hsinchu, 30077 Taiwan; 4 Neutron Science Section, Japan Proton Accelerator Research Complex, Japan Atomic Energy Agency, Tokai-mura, Ibaraki, 319-1195 Japan; 5 Neutron Science and Technology Center, Comprehensive Research Organization for Science and Society, Tokai-mura, Ibaraki, 319-1106 Japan

**Keywords:** Magnetic properties and materials, Structure of solids and liquids, Magnetic properties and materials, Structure of solids and liquids

## Abstract

In condensed matter physics, pressure is frequently used to modify the stability of both electronic states and atomic arrangements. Under isotropic pressure, the intermetallic compound MnP has recently attracted attention for the interplay between pressure-induced superconductivity and complicated magnetic order in the vicinity . By contrast, we use uniaxial stress, a directional type of pressure, to investigate the effect on the magnetism and crystal structure of this compound. An irreversible magnetisation response induced by uniaxial stress is discovered in MnP at uniaxial stress as low as $0.04\ \text {GPa}$. Neutron diffraction experiments reveal that uniaxial stress forms crystal domains that satisfy pseudo-rotational symmetry unique to the MnP-type structure. The structure of the coexisting domains accounts for the stress-induced magnetism. We term this first discovered phenomenon atomic reconstruction (AR) induced by uniaxial stress. Furthermore, our calculation results provide guidelines on the search for AR candidates. AR allows crystal domain engineering to control anisotropic properties of materials, including dielectricity, elasticity, electrical conduction, magnetism and superconductivity. A wide-ranging exploration of potential AR candidates would ensure that crystal domain engineering yields unconventional methods to design functional multi-domain materials for a wide variety of purposes.

## Introduction

Application of pressure can directly reduce interatomic distance, stabilizing exotic phases of solids. As one of the most outstanding cases, the observation of room-temperature superconductivity in $\text {LaH}_{10}$ under pressure of approximately $170\, \text {GPa}$ has attracted great interest in terms of the effects of ultrahigh pressure on electronic states^1^. However, to bring about novel phenomena, not only the magnitude of pressure is important, but also its direction, because a crystal has unique orientations. Application of small uniaxial stress enables control of and can even induce multiferroic characteristics. Uniaxial stress as low as about $0.03\, \text {GPa}$ $(30\, \text {MPa})$ tuned a population of conjugate ferroelectric domains to change the value of nett electric polarisation in geometrically frustrated magnet $\text {CuFe}_{1-x}\text {Ga}_{x}\text {O}_{2}$^2^. When the stress was increased to around $1\,\, \text {GPa}$ and a magnetic field was simultaneously applied, pure $\text {CuFeO}_{2}$ exhibited an unconventional spin-driven ferroelectric phase^3^. This mechanical stimulus, so-called “anisotropic pressure,” is distinct enough from isotropic pressure to be classified as an independent parameter that realises unique thermodynamic phases.

**Figure 1:**
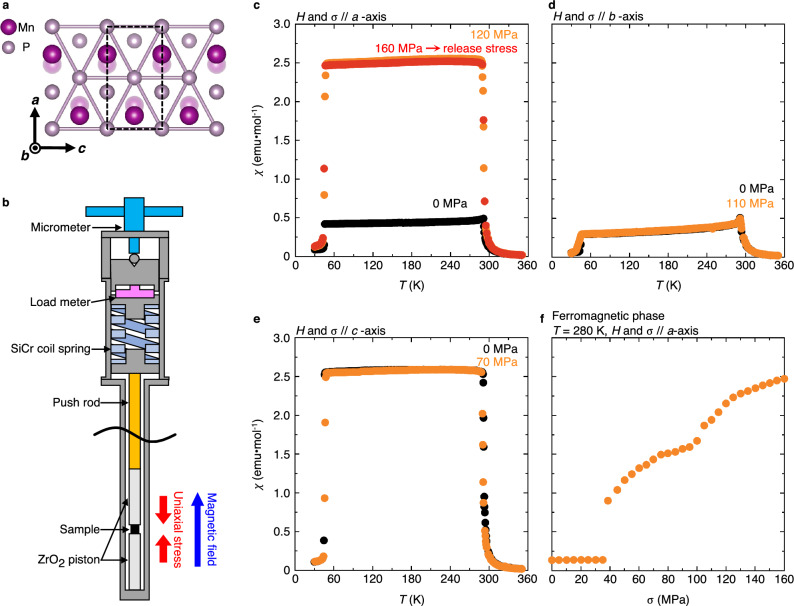
Magnetic susceptibility of MnP under conditions of uniaxial stress. (**a**) Crystal structure of MnP featuring distorted hexagonal structure of P bonds. The dashed rectangle represents the orthorhombic unit cell. (**b**) Schematic of a uniaxial stress cell used in magnetisation measurements. See Methods for more details about the individual modules mentioned here. (**c**–**e**) Temperature dependence of magnetic susceptibility for samples at ambient pressure (pristine sample, black circles), under uniaxial stress (orange circles) and after applied uniaxial stress along *a*-axis is released (stress-released sample, red circles). (**f**) Magnetic susceptibility along *a*-axis in the ferromagnetic phase under increasing uniaxial stress along *a*-axis.

Because of the wide potential of its interesting pressure-induced responses^4–13^, manganese phosphide (MnP) is an intriguing material with complex magnetic ordering: in zero magnetic field, a ferromagnetic phase below $T_{\text {C}}=291\text { K}$ and a double helical phase below $T_{\text {S}}\approx 50\,\text {K}$^14,15^. In 2015, the first case of Mn-based superconductivity was found in MnP below $T_{\text {SC}}\approx 1\,\text { K}$ when $8\,\, \text {GPa}$ isotropic pressure was applied^4^. On the other hand, uniaxial stress reorganised distribution of magnetic chirality domains in MnP, i.e., the left-handed and right-handed spiral arrangements of spins^16^. These observations suggest that MnP may have further striking responses to pressure yet to be discovered. Here, we perform magnetisation, magnetic susceptibility and neutron diffraction measurements on MnP single crystals under several conditions of uniaxial stress. Note that the directions of magnetic fields *H* and uniaxial stress $\sigma$ are always based on orientations of the samples before the fields or stress is applied. Figure 1a illustrates crystal structure of MnP. We adopt the orthorhombic unit cell with the lattice parameters $a=5.91$ Å $>b=5.26$ Å $>c=3.17$ Å (space group *Pbnm*) at room temperature^17^. In the ferromagnetic phase, the magnetic easy axis is *c*-axis, and the hard and intermediate directions are *a*-axis and *b*-axis, respectively. For clarity, we use the expressions “pristine sample” and “stress-released sample” for, respectively, samples under ambient pressure and after applied uniaxial stress along *a*-axis is released, in this Article and Supplementary Information (see Methods for more details).

Figure 1c–e show temperature dependence of magnetic susceptibility for several MnP cuboid samples under conditions of uniaxial stress, which we apply with a stick-type piston-cylinder stress cell illustrated in Fig. 1b. A significant increase of magnetic susceptibility along *a*-axis is induced by uniaxial stress along *a*-axis in the ferromagnetic phase, while magnetic susceptibility along *b*- and *c*-axes does not exhibit large changes under uniaxial stress along *b*- and *c*-axes, respectively. Note that the increase holds in stress-released sample; the stress-induced state of MnP remains stable even after the applied stress is released. We refer to the fact that the transition temperature $T_{\text {C}}$ remains largely unchanged. This suggests that no magnetic properties change between pristine sample and stress-released sample. Figure 1f shows magnetic susceptibility along *a*-axis in the ferromagnetic state under various uniaxial stress along *a*-axis. There exists threshold stress $\sigma \approx 40\,\text { MPa}$, above which the values rise with increasing stress. The shoulder-like slope is presumably ascribable to inhomogeneous formation of crystal domains with some kind of irreversible structural changes (see Supplementary Note 1 for further details). Note that magnetic susceptibility along *a*-axis at $120\,\text { MPa}$ does not match between Fig. 1c and f. The discrepancy implies sample-to-sample variation in volume fractions of the stress-induced crystal domains. We believe that the volume fractions depend on the accuracy of *a*-axis. As we adjust crystal orientations of pristine samples by hand during sample preparation, their *a*-axes randomly deviate by a few degrees from those we expected, which leads to deviation of the direction of uniaxial stress from the *a*-axes. Thus, a proportion of the stress-induced crystal domains is a function of the deviation.

**Figure 2:**
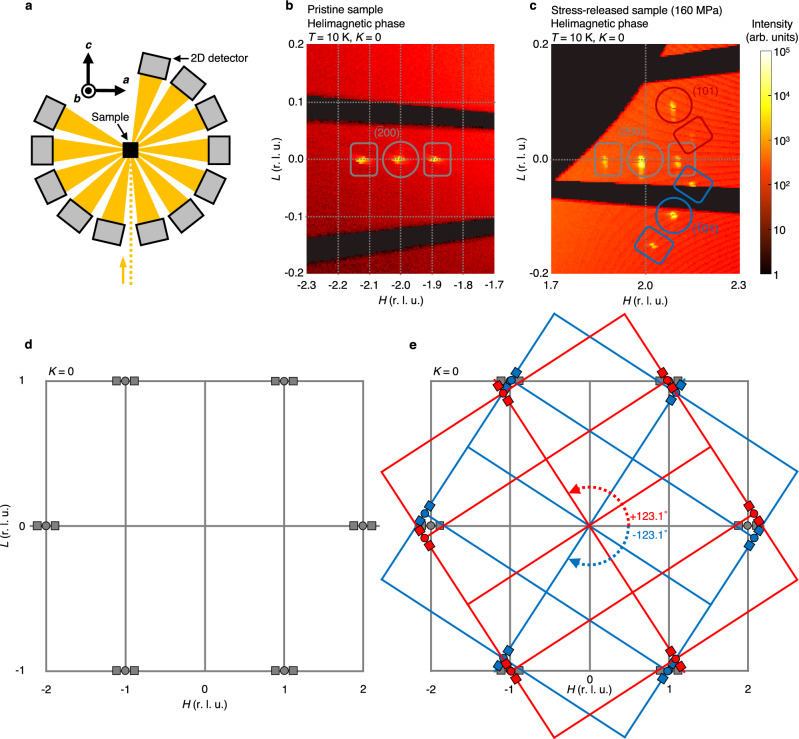
Time-of-flight neutron diffraction measurements of pristine sample and stress-released sample. (**a**) Schematic of a top view of the experimental setup of time-of-flight neutron diffraction. The dotted orange line and orange areas denote flight paths of the incident and diffracted neutrons, respectively. Note that the black arrows with the symbols represent crystal axes of pristine sample. (**b**, **c**) Contour maps of neutron intensity in (*H*0*L*) planes of reciprocal lattice space in the helimagnetic phase observed for pristine sample (**b**) and stress-released sample (**c**). The released uniaxial stress along *a*-axis is $160\, \text {MPa}$. Circles and rectangles with indices denote nuclear reflection and helimagnetic satellite reflection, respectively. Colours represent the crystal domain to which the reflection and indices belong. The scale of **c** is determined based on the reciprocal lattice vectors of the original domain (grey). (**d**, **e**) Schematic of (*H*0*L*) planes of reciprocal lattice space for pristine sample (**d**) and stress-released sample (**e**) in the helimagnetic phase. Circles and rectangles denote nuclear reflection and helimagnetic satellite reflection, respectively. The colour code is the same as in **b** and **c**. The dashed arrows show rotation angles of crystal axes of the stress-induced domains (red, blue) with respect to those of the original domain (grey).

**Figure 3:**
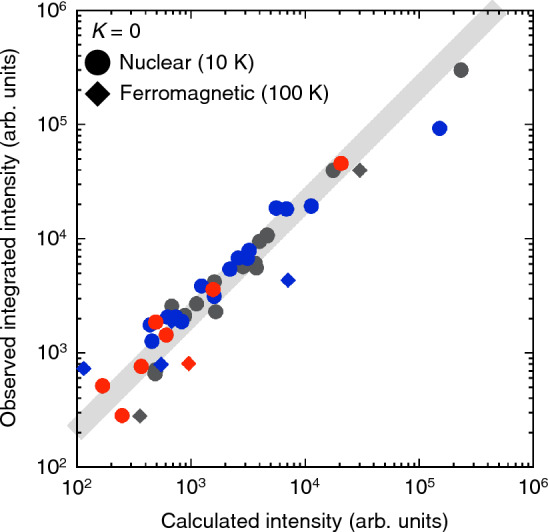
Observed neutron integrated intensity of the (*H*0*L*) nuclear and ferromagnetic reflection for stress-released sample plotted against calculated values for the nuclear and ferromagnetic peaks. Circles and rhombuses denote nuclear and ferromagnetic reflection, respectively. The colour code is the same as in Fig. 2b and c. The volume fractions of the original domain (grey) and stress-induced domains (red, blue) are $46.1\%$, $5.5\%$ and $48.4\%$, respectively, included in the calculated values. The solid grey line is a guide for the eyes. The error bars representing standard deviations are small enough to be covered with the symbols. The released uniaxial stress along *a*-axis is $160\, \text {MPa}$. See Supplementary Note 3 for details about how we obtain integrated intensities of nuclear and ferromagnetic reflection.

To identify a structural cause of the irreversible magnetic responses, we perform time-of-flight neutron diffraction measurements at $10\, \text {K}$, $100\, \text {K}$ and $320\, \text {K}$ for pristine sample and several stress-released samples using the SENJU diffractometer installed at the MLF, J-PARC (we also obtain preliminary data using the Sika spectrometer installed at ANSTO. See Methods for more details about the neutron diffraction experiments in the Sika spectrometer and SENJU diffractometer, and Supplementary Note 2 for the experimental results in the Sika spectrometer). Note that the samples used in neutron diffraction measurements are not identical to the samples used in magnetisation measurements; in principle, we need at least two pristine samples to investigate both the structural changes and magnetic responses. Uniaxial stress along *a*-axis is applied and then released only once on one sample. The amounts of the released stress are $30\text { MPa}$, $60\text { MPa}$, $80\text { MPa}$, $160\text { MPa}$ and $200\text { MPa}$. As shown in Fig. 2a, we mount a cuboid sample with the (*H*0*L*) scattering plane. The sample is rotated around *b*-axis so that we can have access to as many peaks as possible with the two-dimensional detectors. Figure 2b and c show part of (*H*0*L*) planes of reciprocal lattice space in the helimagnetic phase observed for pristine sample and stress-released sample with the released stress of $160\text { MPa}$. For pristine sample, $({\bar{2}} 0 0)$ nuclear reflection is observed, neighboured by a pair of satellite reflection due to the helical magnetic order with the propagation wave vector ${\varvec{q}}\approx (0.11,0,0)$^15,18,19^. For stress-released sample, on the other hand, some reflection along with its satellites is also observed that belongs to specific crystal domains of MnP without significant structural phase transformations or lattice distortions. This result indicates that uniaxial stress along *a*-axis irreversibly induces crystal domains in the single-domain crystal. Here, we use the term “stress-induced domains” for the two induced crystal domains with different orientations from those of pristine sample. The remaining part of the single crystal is referred to as “original domain.” Figure 2d and e illustrate schematics of (*H*0*L*) planes of reciprocal lattice space for pristine sample and stress-released sample in the helimagnetic phase. The crystal axes of the stress-induced domains are rotated by $\pm 123.1^\circ \pm 0.2^\circ$ (double sign in arbitrary order) around the shared *b*-axis with respect to those of the original domain. All the (*H*0*L*) nuclear and ferromagnetic reflection observed for stress-released sample is successfully indexed based on coexistence of the three crystal domains, but not based on a single-domain crystal (see Fig. 3). Neutron diffraction measurements for the released stress up to $200\text { MPa}$ indicate that the stress-induced domains are formed at $\sigma \approx 40\text { MPa}$, which corresponds to the value where magnetic susceptibility along *a*-axis begins to increase as shown in Fig. 1f (see Supplementary Note 4 for the neutron diffraction results corresponding to Fig. 2c). X-ray Laue backscattering patterns reveal that the multi-domain structure persists and does not revert to the single-domain structure even if stress-released sample is annealed until its surface is oxidised. According to observation of X-ray Laue diffraction peaks (see Supplementary Note 5 for more details), we believe that formation of the stress-induced domains is a totally different phenomenon from plastic deformations as well as structural phase transformations. Therefore, we term it “AR induced by uniaxial stress.” To the best of our knowledge, no such structural change as AR has yet been reported for any material.

We next discuss the origin of AR in MnP by noting the fact that the relative rotation angles of the stress-induced domains deviate from $\pm 120^\circ$. This can be attributed to the existence of pseudo-symmetry unique to the MnP-type structure, as explained below. We can regard the MnP-type structure as the hexagonal NiAs-type structure (space group $P6_{3}/mmc$) having a slight elongation along *a*-axis of MnP^17^; at room temperature, the ratio *a*/*c* is 1.86 for MnP, which does not possess three-fold rotational symmetry, while it is exactly $\sqrt{3}$ for the NiAs-type structure, where the symmetry is preserved. Therefore, the atomic arrangement of the MnP-type structure should be nearly unchanged even if we rotate it by almost $\pm 120^\circ$ around *b*-axis of MnP. The two operations, in analogy to the three-fold symmetry of the NiAs-type structure, correspond to rotating the rectangular cell in Fig. 1a around *b*-axis so that either of its two diagonals overlaps before and after the rotation. The ratio *a*/*c* for MnP can be used to calculate the rotation angle $\theta _{\text {AR}}$:

1 $$\begin{aligned} \theta _{\text {AR}}=\pm 2\arctan \left( \frac{a}{c}\right) \approx \pm 124^\circ , \end{aligned}$$

not $\pm 120^\circ$ because of the deviated symmetry of the MnP-type structure, which we call pseudo-orthohexagonal symmetry (POHS), while the symmetry is orthohexagonal for the NiAs-type structure. The crystal orientations determined from the calculated angles well correspond to the stress-induced domains, implying that in MnP, AR occurs between atomic arrangements that satisfy POHS.

**Figure 4:**
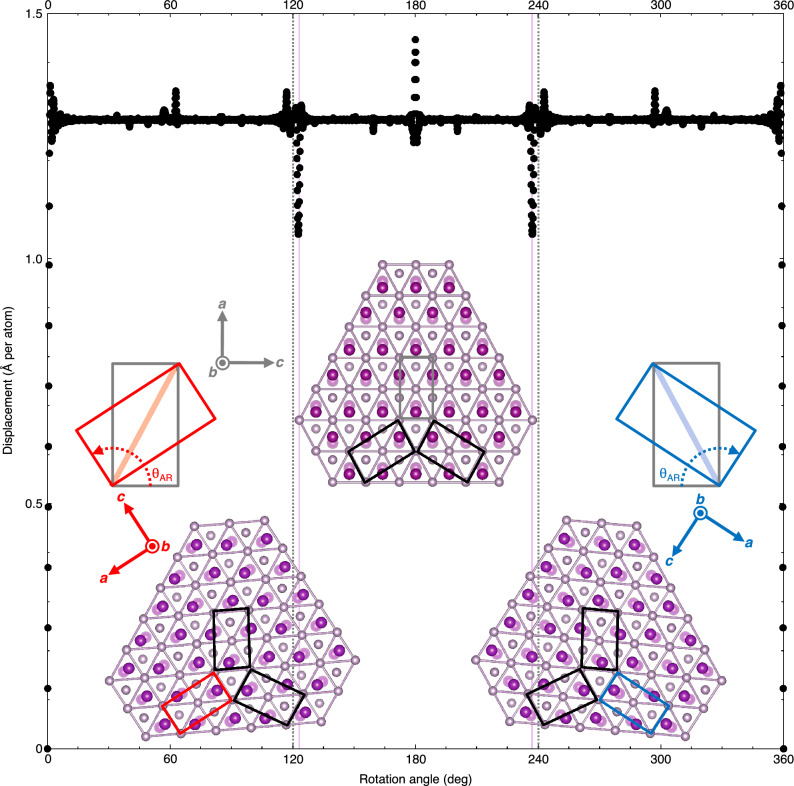
Rotation angle dependence of calculated actual atomic displacement due to rotation around *b*-axis in MnP. The positive direction of the rotation is anticlockwise around a fixed rotation axis. The dotted grey lines highlight positions of the rotation angles $120^\circ$ and $240^\circ$, while the solid pink ones highlight positions of the angles $123^\circ$ and $237^\circ$. Inset shows the crystal structure satisfying POHS with the corresponding operations of rotation by $\theta _{\text {AR}}$. Grey, red and blue quadrilaterals represent the orthorhombic unit cells, while black ones represent the monoclinic apparent cells described in the main text. See Methods for further details on how we choose the rotation axis and calculate the displacement of each atom.

To confirm the existence of POHS, we consider actual atomic displacements due to crystal rotation, which can be an indicator of rotational symmetry and pseudo-rotational symmetry of the structure: if a crystal has certain rotational symmetry around a fixed axis, the corresponding symmetry operations generate zero displacement. If, conversely, a crystal has certain pseudo-rotational symmetry around an axis, operations involving rotation around that axis should lead to small but significant displacements. Here, we emphasise the sense of “actual” atomic displacements; it does not refer to the arced orbits of each atom but the spacing between atoms of the same element before and after crystal rotation. Figure 4 shows calculation of the actual atomic displacements due to rotation around *b*-axis in MnP. Sharp depressions with local minimums of approximately 1 Å appears at $123^\circ$ and $237^\circ$ ($360^\circ -123^\circ$), not $120^\circ$ and $240^\circ$ ($360^\circ -120^\circ$). This indicates that a nontrivially minimum atomic displacement is needed for an ambient MnP single crystal to transform into the same structure with other orientations that follow POHS. As shown in the inset of Fig. 4, on a microscopic level, the structural change means deformations of the orthorhombic unit cell to a monoclinic apparent cell, and vice versa. The computational work strongly suggests the possibility that POHS gives rise to AR in MnP.

**Figure 5:**
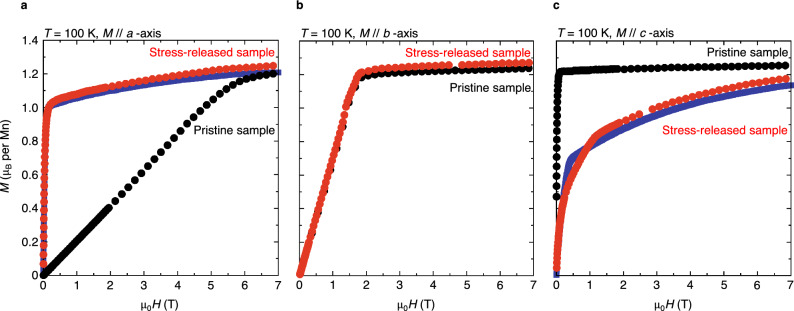
Magnetisation curves of pristine sample and stress-released sample at 100 K. Black and red circles denote the measurements of pristine sample and stress-released sample, respectively. The released uniaxial stress along *a*-axis is $160 \ \text {MPa}$. In **a** and **c**, the solid blue lines are the fitted model curves with a volume fraction of the original domain $4.2\%$ (see Methods).

Stress-released sample has the structure of the coexisting crystal domains. Thus, we should be able to express magnetisation of stress-released sample as a linear combination of magnetisation of the original domain and stress-induced domains with their volume fractions as coefficients. To confirm the multi-domain structure by means of magnetisation measurements, we prepare model curves composed of magnetisation of pristine sample in the direction of *a*- (*c*-) axis and $120^\circ$ from *a*- (*c*-) axis around *b*-axis, and then fit them to magnetisation of stress-released sample along *a*- (*c*-) axis (see Methods for further details). Note that when fitting, we cannot distinguish the contributions of the stress-induced domains because of geometrical symmetry between the direction of the magnetic field and two *a*- (*c*-) axes of the stress-induced domains. The fitting is performed above $1\text { T}$ to avoid effects of demagnetizing fields on the accuracy of the estimated volume fraction of the original domain; in ferromagnets, magnetisation curves at low fields are very sensitive to demagnetizing effects. As shown in Fig. 5, the model curves along *a*- and *c*-axes well describe the stress-induced magnetism where the easy and hard axes seem to be reversed. Magnetisation along *b*-axis remains largely unchanged, which is consistent with the multi-domain structure because the original domain shares its *b*-axis with the stress-induced domains.

Our observation of the irreversible magnetic responses induced by uniaxial stress in MnP opens new areas of study in both theoretical and applied condensed matter physics. The fundamental cause for the stress-induced magnetism in MnP is AR, i.e., formation of the crystal domains that exhibit the pseudo-rotational symmetry unique to the MnP-type structure. Thus, other MnP-type crystals with a *a*/*c* value close to $\sqrt{3}$ have tremendous potential for AR. We believe that they are not the only cases, though; AR could also be induced by uniaxial stress in a specific direction if a crystal has certain pseudo-rotational symmetry, regardless of whether its foldness is forbidden in periodic crystals. Still, the detailed mechanism of AR remains a mystery. It would be interesting to examine how uniaxial stress takes part in the unknown process where pseudo-symmetry plays a crucial role. At the applied level, we have demonstrated that AR can serve as a basis for crystal domain engineering, techniques to adjust anisotropic properties of materials, such as dielectricity, elasticity, electrical conduction, magnetism and superconductivity, by introducing particular crystal domains into a single-domain crystal. As exemplified by the case of MnP, a sample tailored with AR has a blend of the anisotropic responses inherent in a single-crystal sample. The blend ratio corresponds to volume fractions of the crystal domains. Hence, the use of AR would enable modulation of the intrinsic features of materials without necessitating any complex physical processes. An extensive exploration of potential candidates for AR would ensure that crystal domain engineering yields unconventional methods to design functional multi-domain materials for a wide variety of purposes.

## Methods

### Sample preparation

Single crystals of MnP were grown by the Bridgman method from a mixture of stoichiometrically equal amounts of Mn and P powder. The crystal axes were determined from the X-ray diffraction patterns. Several cuboid samples with typical dimensions of $2\times 2\times 2\, \text {mm}^3$ were prepared from the single crystals. We performed magnetisation, magnetic susceptibility, and neutron diffraction measurements on MnP samples under some or all of the following three conditions with respect to uniaxial stress: at ambient pressure, under uniaxial stress, and after applied uniaxial stress was released. As introduced in the main text, the first and third cases in the direction of *a*-axis correspond to pristine sample and stress-released sample, respectively.

### Magnetisation and magnetic susceptibility measurements

Magnetisation and magnetic susceptibility measurements were performed using a commercial superconducting quantum interference device magnetometer (MPMS-XL7AC: Quantum Design). We used the stick-type piston-cylinder stress cell which could be inserted into the magnetometer^20^ to apply uniaxial stress on MnP samples (see Fig. 1b). Mechanical force was applied and tuned by rotating the micrometer, to press the load meter and SiCr coil spring together. The load meter monitored changes of the internal force moderated by the spring. Uniaxial compressive stress was introduced to a cuboid sample by a combination of the push rod and the $\text {ZrO}_{2}$ pistons sandwiching the sample. The temperature ranged from $280\, \text {K}$ to $330\, \text {K}$ while uniaxial stress was applied to reach the target values.

### Neutron diffraction experiments

A preliminary neutron diffraction experiment was performed using the cold-neutron triple-axis spectrometer Sika at the Australian Centre for Neutron Scattering (ACNS) of the Australian Nuclear Science and Technology (ANSTO) facility^21,22^. The incident and scattered neutron energies were fixed at $14.87\, \text {meV}$.

A neutron diffraction experiment was performed using the time-of-flight single crystal neutron diffractometer SENJU installed at the Materials and Life Science Experimental Facility (MLF), Japan Proton Accelerator Research Complex (J-PARC)^23^. A neutron wavelength of 0.4–4.4 Å was used. To prepare several samples for the neutron diffraction measurements, MnP single crystals were cut to cuboid shapes with dimensions of $(1.5\sim 2.3)\times (1.5\sim 2.5)\times (1.2\sim 2.1)\text { mm}^3$ and mass of $15.3\text { mg}$ to $68.1\, \text {mg}$. We applied and then released uniaxial stress along *a*-axis on the samples at room temperature with a tabletop uniaxial stress cell whose mechanism is essentially the same as that described in the previous subsection. The observed reciprocal lattice maps, time-of-flight profiles, integrated intensities were output by the software STARGazer^24^.

### Calculations

The calculation of the neutron scattering intensities and atomic displacements, and model curve fitting were performed using the software Mathematica (https://www.wolfram.com/mathematica).

To calculate the atomic displacements due to rotation of a MnP crystal, we adopted the fractional coordinates of MnP^17^. The lattice parameters used in this calculation are $a=5.8628$ Å, $b=5.2487$ Å and $c=3.2009$ Å, the values averaged over the measurements of the original domain at $10\, \text {K}, 100\, \text {K}$ and $320\, \text {K}$ in the neutron diffraction experiment with the SENJU diffractometer. The targeted space of the real lattice was confined within a cylinder of radius 100 Å and height 1*b*. The axis of the cylinder is parallel to *b*-axis and matches the rotation axis. The two-dimensional coordinates of a certain P atom, such as the intersection of the diagonals of the rectangular cell in Fig. 1a, are fixed on the rotation axis. This constraint is intended to ensure that the calculation reflects the distorted hexagonal structure of P bonds. The atomic displacement per atom was calculated by taking the root-mean-square of the shortest interatomic distance between the same kind of atoms before and after crystal rotation. In order to simplify the code, we used the Nearest function (https://reference.wolfram.com/language/ref/Nearest), which linked some atoms to the same destination. However, the effect on the result is negligibly small.

The model curves along *a*- and *c*-axes, $M^{a}$ and $M^{c}$, respectively, were fitted above $\mu _{0}H=1\, \text {T}$ simultaneously in the least-squares method to the magnetisation curves of stress-released sample along *a*- and *c*-axes shown in Fig. 5a and c:

2 $$\begin{aligned} M^{a}(H)=v_{\text {org}}M_{\text {pristine}}^{a}(H) +(1-v_{\text {org}})M_{\text {pristine}}^{a+120^\circ }(H), \end{aligned}$$

3 $$\begin{aligned} M^{c}(H)=v_{\text {org}}M_{\text {pristine}}^{c}(H) +(1-v_{\text {org}})M_{\text {pristine}}^{c+120^\circ }(H), \end{aligned}$$

where $v_{\text {org}}$ is a parameter representing a volume fraction of the original domain, $M_{\text {pristine}}^{a}$, $M_{\text {pristine}}^{c}$, $M_{\text {pristine}}^{a+120^\circ }$ and $M_{\text {pristine}}^{c+120^\circ }$ are magnetisations of pristine sample in the direction of *a*- and *c*-axes, and $120^\circ$ from *a*- and *c*-axes around *b*-axis, respectively. To prepare continuous functions for $M_{\text {pristine}}^{a}$, $M_{\text {pristine}}^{c}$, $M_{\text {pristine}}^{a+120^\circ }$ and $M_{\text {pristine}}^{c+120^\circ }$, we interpolated the measurements of pristine sample in Fig. 5a, c and Supplementary Fig.  6 using the Hermitian interpolation method.

### Crystal structure images

To visualise the crystal structure images, we used the Visualization for Electronic and STructural Analysis (VESTA)^25^.

### Supplementary Information

Supplementary Information.

## Data Availability

All data supporting the present work are available from the corresponding author upon reasonable request.

## Code availability

All Mathematica codes used to generate the results presented in the paper are available from the corresponding author upon reasonable request.
