# Supplementary material for: Atomic reconstruction induced by uniaxial stress in MnP
Source: Sci Rep. 2023 Aug 23;13:13750. doi: 10.1038/s41598-023-40806-1 (PMC10447523; doi:10.1038/s41598-023-40806-1)
Supplement: Supplementary file 1 — Supplementary Information. [file 41598_2023_40806_MOESM1_ESM.pdf]

# Atomic reconstruction induced by uniaxial stress in MnP

**Tatsuya Kozawa[1,*], Masayoshi Fujihala[1,2,†], Takeru Uchihara[1], Setsuo Mitsuda[1,‡], Shin-ichiro Yano[3], Hiromu Tamatsukuri[1,4], Koji Munakata[5] and Akiko Nakao[5]**

[1]Department of Physics, Faculty of Science, Tokyo University of Science, Shinjuku, Tokyo 162-8601, Japan

[2]Advanced Science Research Center, Japan Atomic Energy Agency, Tokai-mura, Ibaraki 319-1195, Japan

[3]National Synchrotron Radiation Research Center, Hsinchu 30077, Taiwan

[4]Neutron Science Section, Japan Proton Accelerator Research Complex, Japan Atomic Energy Agency, Tokai-mura, Ibaraki 319-1195, Japan

[5]Neutron Science and Technology Center, Comprehensive Research Organization for Science and Society, Tokai-mura, Ibaraki 319-1106, Japan

[*]1221519@alumni.tus.ac.jp

[†]fujihala@post.j-parc.jp

[‡]mitsuda@rs.tus.ac.jp

# Supplementary information

## I. Surfaces of pristine sample and stress-released sample

As seen in Fig. S1, light white lines appeared along *b*-axis on the surface of stress-released sample. We believe that contrast between the dark green and light white lines indicates boundaries separating nonidentical crystal structure. We also conclude that inhomogeneous stress inside the cuboid sample induced a set of structural changes that in turn caused a stepwise increase in magnetic susceptibility along *a*-axis (see Fig. 1f in the main text), and that generated random stripe-like patterns on the surface. MnP single crystals polished into cuboids held their shapes up to uniaxial stress along *a*-axis of at least 200 MPa.

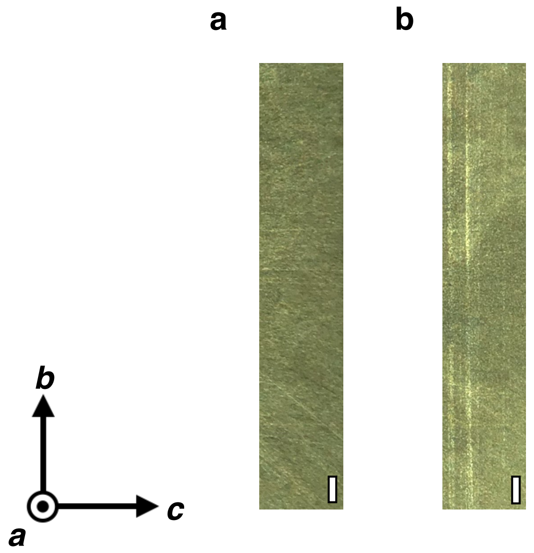


**Fig. S1.** Optical microscope images of surfaces of pristine sample (**a**) and stress-released sample (**b**) using unpolarised light. The two images show the same area of the identical sample at a resolution of 72 ppi. The black arrows with the symbols denote crystal axes of pristine sample. The released uniaxial stress along *a*-axis is 100 MPa. Scale bars: 0.1 mm.

## II. Preliminary neutron diffraction experiment

A preliminary neutron diffraction experiment was performed using the Sika spectrometer installed at the ANSTO. Fig. S2 shows rocking curves for pristine sample and stress-released sample. The experimental results suggest that uniaxial stress along *a*-axis irreversibly induces crystal structure which seems to possess six-fold symmetry around *b*-axis of pristine sample.

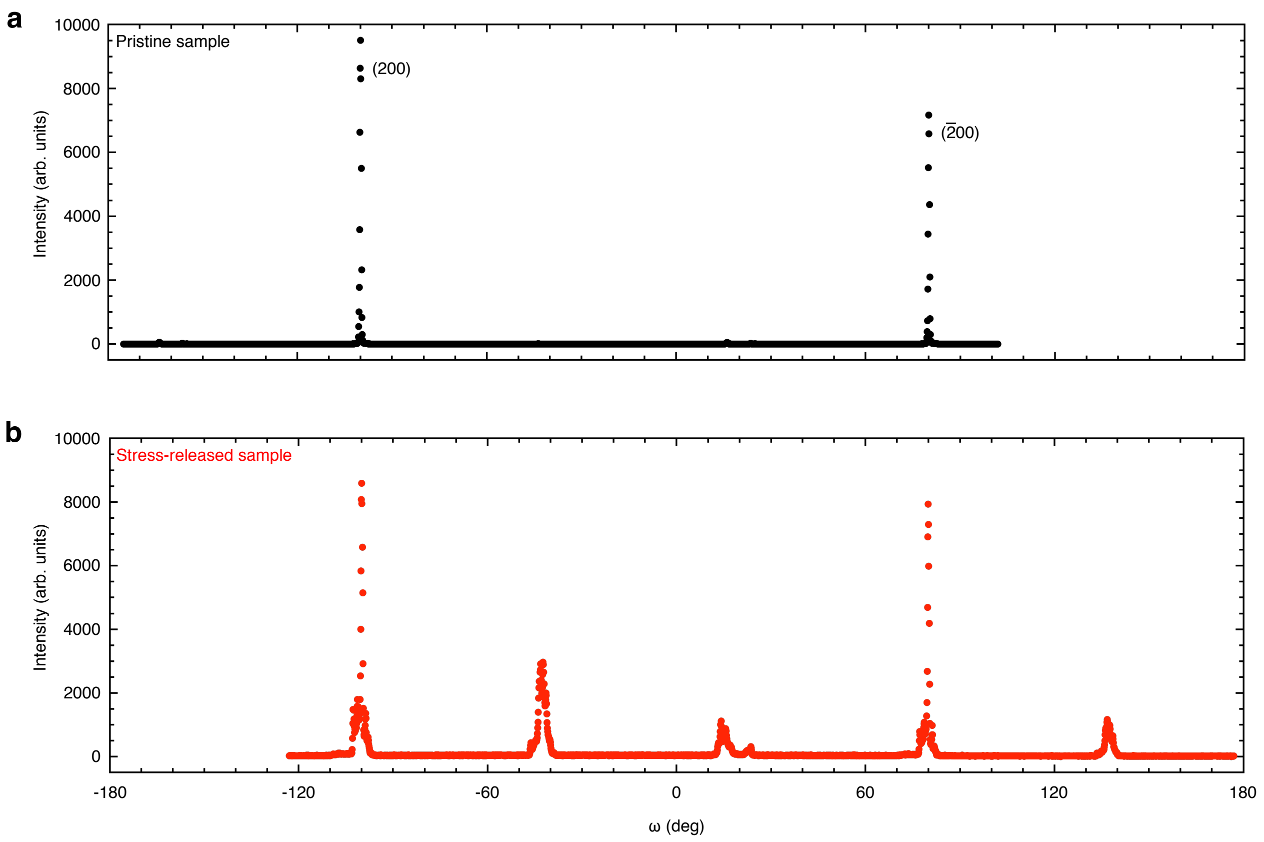


**Fig. S2.** Rocking curves for (200) reflection in (*H*0*L*) planes of pristine sample (**a**) and stress-released sample (**b**) at room temperature. The origins of the rotation angle $\omega$ in **a** and **b** are chosen arbitrarily. In **b**, the scattering angle $2\theta$ for the (200) reflection is determined based on the peak positions of the identical sample before uniaxial stress is applied along *a*-axis. The released stress is 100 MPa.

## III. Neutron diffraction intensities of nuclear and ferromagnetic reflection

As for observation of neutron diffraction intensity, peaks of magnetic reflection due to ferromagnetic order always overlap those of nuclear reflection. Therefore, we need to subtract intensities of nuclear reflection from total intensities observed in the ferromagnetic phase to get intensities of ferromagnetic reflection.

We obtained integrated intensities at 10 K (helimagnetic phase) and 100 K (ferromagnetic phase), $I_{obs}(T = 10$ K) and $I_{obs}(T = 100$ K), respectively, by processing the data taken in the SENJU diffractometer with the software STARGazer [1]. The observed integrated intensities of nuclear and ferromagnetic reflection, $I_{obs}$(nuclear) and $I_{obs}$(ferromagnetic), respectively, were obtained by the following equations:

$$I_{obs}(\text{nuclear}) = I_{obs}(T = 10\text{ K}) \quad \text{(S1)}$$

$$I_{obs}(\text{ferromagnetic}) = I_{obs}(T = 100\text{ K}) - I_{obs}(T = 10\text{ K}) \quad \text{(S2)}$$

At 320 K (paramagnetic phase), intensities of nuclear reflection are attenuated by the effects of thermal vibrations known as the Debye-Waller factor. At 10 K, where MnP has the helical magnetic structure in zero magnetic field, not the conical one, only nuclear reflection exists at Bragg points (with integer indices), while magnetic reflection appears not at the Bragg points, but at the satellite points on both sides of them. Therefore, we adopted the integrated intensities at 10 K (except the helimagnetic satellite reflection), not at 320 K, as $I_{obs}$(nuclear). The values of $I_{obs}$(nuclear) well corresponded to our calculation results.

As an example of the ferromagnetic and helimagnetic satellite reflection, Fig. S3 shows time-of-flight profiles including (-2 0 0) reflection for pristine sample at 10 K and 100 K. There exists finite $I_{obs}$(ferromagnetic) at (-2 0 0), where nonzero intensity of ferromagnetic reflection was expected according to our calculation.

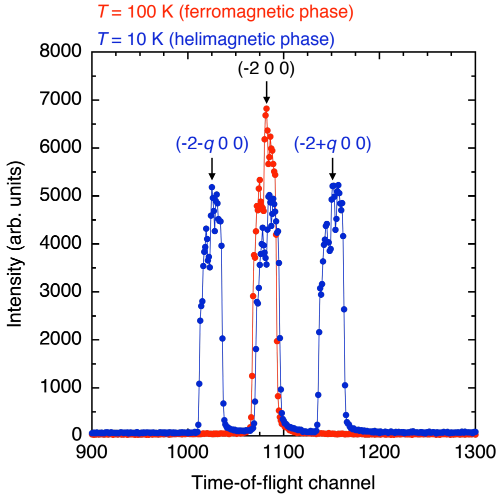


**Fig. S3.** Time-of-flight profiles including (-2 0 0) reflection for pristine sample at 10 K (helimagnetic phase) and 100 K (ferromagnetic phase). Time-of-flight channels correspond to magnitude of scattering vectors in one specific direction. The number $q$ stands for the propagation wave number of the helical magnetic order.

As for calculation of neutron diffraction intensity, the fractional coordinates of MnP [2] was used to obtain calculated intensities of nuclear and ferromagnetic reflection. We adopted the value 1.33 $\mu_B$ as the magnetic moment of Mn along $c$-axis in the ferromagnetic state [3].

## IV. Neutron diffraction measurements for several stress-released samples

We performed time-of-flight neutron diffraction measurements at 10 K, 100 K and 320 K for stress-released samples with various released uniaxial stress along $a$-axis using the SENJU diffractometer installed at the MLF, J-PARC. Fig. S4 shows part of ($H0L$) planes of reciprocal lattice space in the paramagnetic phase observed for several stress-released samples with the released stress of $\sigma$ = 30, 60, 80, 200 MPa. Nuclear reflection from stress-induced crystal domains was observed in the measurements for 60 MPa, 80 MPa and 200 MPa, while only nuclear peaks from a MnP single-domain crystal were observed in the measurements for 30 MPa. The experimental results indicate that the stress-induced domains were formed at $\sigma \approx 40$ MPa. We believe that significant deviation of the direction of uniaxial stress from $a$-axis caused destructive formation of the crystal domains, leading to the powder-like diffraction patterns; another sample with the deviation of about 10° collapsed at 100 MPa.

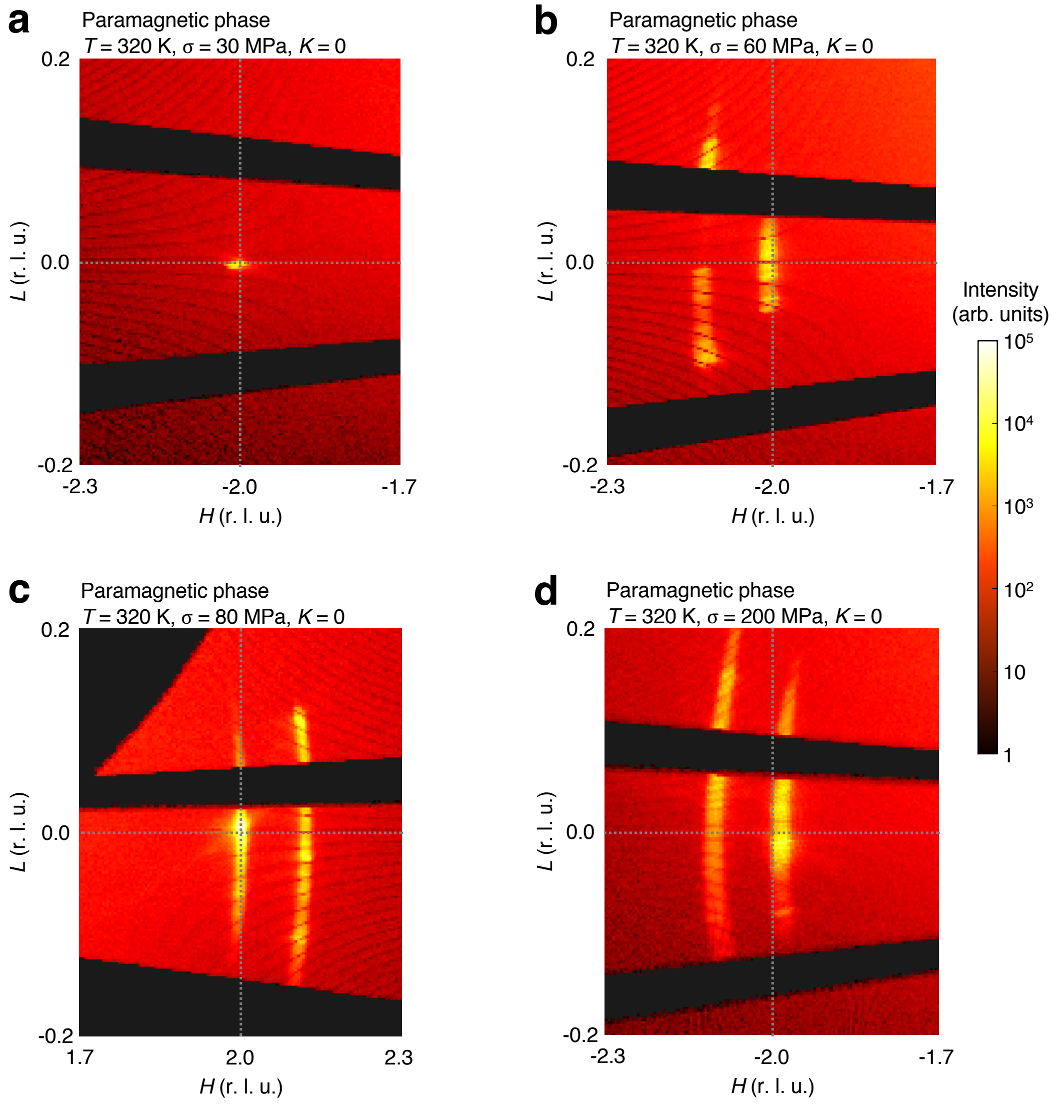


**Fig. S4.** Contour maps of neutron intensity in ($H0L$) planes of reciprocal lattice space in the paramagnetic phase observed for several stress-released samples. These maps correspond to Fig. 2c in the main text. The released uniaxial stress along $a$-axis is 30 MPa (**a**), 60 MPa (**b**), 80 MPa (**c**), 200 MPa (**d**), respectively.

## V. X-ray Laue backscattering patterns of pristine sample and stress-released sample

We observed X-ray Laue backscattering patterns of pristine sample and stress-released sample at room temperature. Fig. S5 shows the Laue patterns in the direction of *a*-axis. The diffraction peaks from stress-released sample were comparable in half width to those from pristine sample, although the pattern of stress-released sample was modified significantly. The experimental result indicates that formation of the stress-induced domains is different from plastic deformations; if it belonged to plastic deformations such as slip deformation, half widths of diffraction peaks from stress-released sample would expand in response to inhomogeneous bends of crystal lattice.

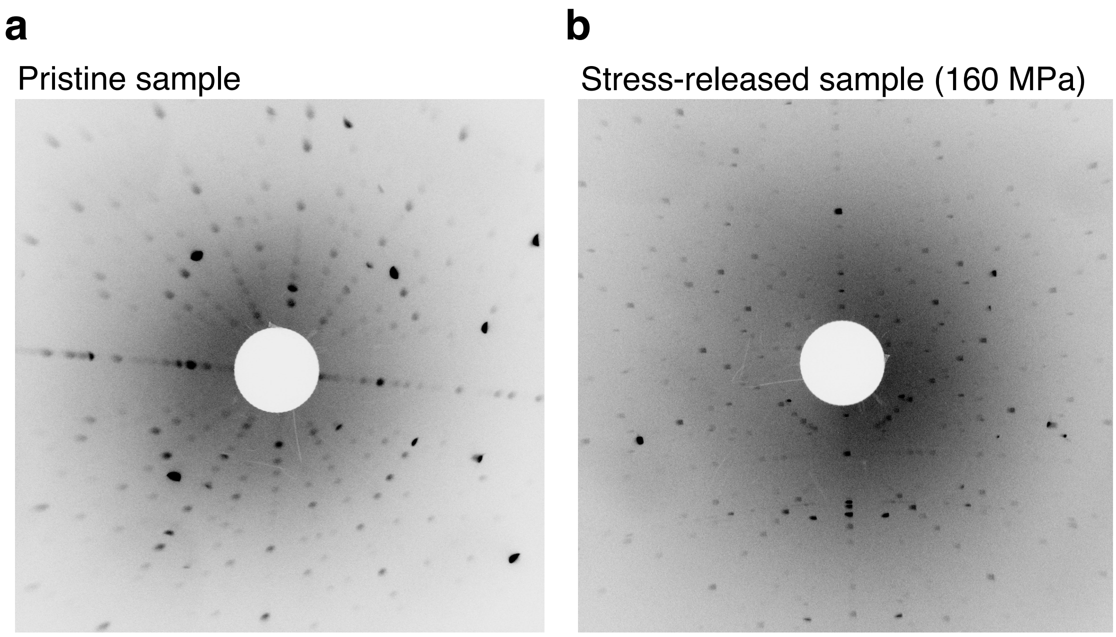


**Fig. S5.** X-ray Laue backscattering patterns of pristine sample (**a**) and stress-released sample (**b**) in the direction of *a*-axis of pristine sample. The released stress along *a*-axis is 160 MPa.

## VI. Magnetisation curves of pristine sample in oblique directions

Fig. S6 shows magnetisation curves of pristine sample at 100 K in the directions 120° from *a*- and *c*-axes around *b*-axis. The measurements were interpolated to obtain continuous functions for $M_{\mathrm{pristine}}^{a+120°}$ and $M_{\mathrm{pristine}}^{c+120°}$ in Eqs. (2) and (3) (see Methods).

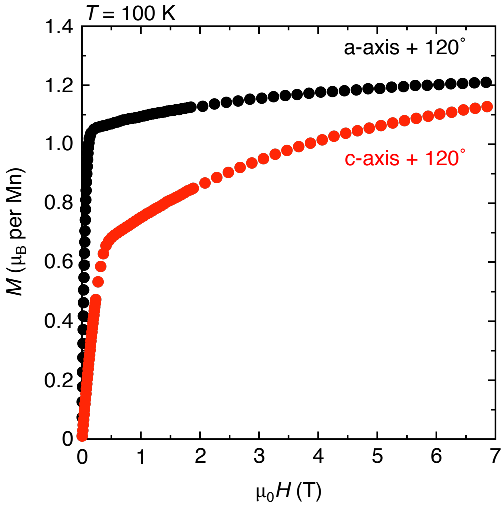


**Fig. S6.** Magnetisation curves of pristine sample at 100 K in the directions 120° from *a*-axis (black circles) and *c*-axis (red circles) around *b*-axis.

## References


[1]. Ohhara, T. *et al*. Development of data processing software for a new TOF single crystal neutron diffractometer at J-PARC. *Nucl. Instrum. Methods Phys. Res. A*. **600**, 195–197 (2009).

[2]. Motizuki, K., Ido, H., Itoh, T. & Morifuji, M. *Electronic Structure and Magnetism of 3d-Transition Metal Pnictides* (Springer Berlin, Heidelberg, 2009).

[3]. Obara, H., Endoh, Y., Ishikawa, Y. & Komatsubara, T. Magnetic Phase Transition of MnP under Magnetic Field. *J. Phys. Soc. Jpn*. **49**, 928–935 (1980).
